# The Use of Medical Grade Honey on Infected Chronic Diabetic Foot Ulcers—A Prospective Case-Control Study

**DOI:** 10.3390/antibiotics12091364

**Published:** 2023-08-24

**Authors:** Adéla Holubová, Lucie Chlupáčová, Jitka Krocová, Lada Cetlová, Linsey J. F. Peters, Niels A. J. Cremers, Andrea Pokorná

**Affiliations:** 1 Faculty of Health and Social Sciences, University of South Bohemia, 370 11 České Budějovice, Czech Republic; adela.holubova@diapodicare.cz; 2 DiaPodi Care, spol. s r.o., 392 01 Soběslav, Czech Republic; diapodicare@seznam.cz; 3 Department of Nursing and Midwifery, Faculty of Health Studies, University of West Bohemia, 301 00 Pilsen, Czech Republic; krocovaj@kos.zcu.cz; 4 Department of Health Sciences, College of Polytechnics Jihlava, 586 01 Jihlava, Czech Republic; lada.cetlova@vspj.cz (L.C.); apokorna@med.muni.cz (A.P.); 5 Triticum Exploitatie BV, Sleperweg 44, 6222 NK Maastricht, The Netherlands; research@mesitran.com; 6 Department of Gynecology and Obstetrics, Maastricht University Medical Centre, 6202 AZ Maastricht, The Netherlands; 7 Department of Health Sciences, Faculty of Medicine, Masaryk University, 625 00 Brno, Czech Republic

**Keywords:** medical grade honey, diabetic foot ulcer, diabetes, anti-inflammatory treatment, glycaemia

## Abstract

Non-healing wounds are usually colonised and contaminated by different types of bacteria. An alternative to antibiotic treatment in patients with infected wounds with local signs of inflammation may be medical grade honey (MGH). MGH has antioxidant, antimicrobial, anti-inflammatory, and immunomodulatory features. This study aims to evaluate the effect of MGH therapy on infected non-healing wounds, especially for diabetic foot syndrome. Prospective, observational case series (*n* = 5) of patients with wounds of diabetic foot syndrome are presented. There were five males with an average age of 61.6 years. All wounds were treated with MGH, and the healing trajectory was rigorously and objectively monitored. In all cases, there was a gradual disappearance of odour, pain, and exudation. Moreover, the wound areas significantly reduced within 40 days and there was a decrease in glycated haemoglobin and glycaemia values. All these outcomes resulted in improved quality of life of the patients. Despite bacterial colonisation, antibiotic treatment was not necessary. All wounds were completely healed. MGH has antimicrobial, anti-inflammatory, and antioxidant effects in diabetic foot syndrome wounds, does not increase glycated haemoglobin or glycaemia levels, and thus constitutes an effective alternative to the use of antibiotics in the treatment of locally infected wounds.

## 1. Introduction

Diabetic foot ulcers (DFUs) are one of the four categories of wounds defined by the Wound Healing Society [1]. A non-healing wound, also called a hard-to-heal or chronic wound, is damage to the skin that heals unusually slowly. Depending on the definition, non-healing wounds are present for at least four weeks as they are locked in the inflammatory state of wound healing, and there are multiple explanations for this arrest, with the theory of persistent infections and exaggerated proteolysis as the most commonly accepted [2]. DFUs are complex, chronic wounds that have a major long-term impact on patient morbidity, mortality, and quality of life (QoL) [3,4,5]. The best-practice guideline “Wound Management in Diabetic Foot Ulcers” describes DFU as infection, ulceration, or destruction of the tissues of the foot (distal to the ankle, including the ankle) in patients with newly diagnosed or previously diagnosed diabetes [5]. The global DFU prevalence is reported at 6.3% (95% CI: 5.4–7.3%), higher in males (4.5%, 95% CI: 3.7–5.2%) than in females (3.5%, 95% CI: 2.8–4.2%), and higher in type 2 diabetic patients (6.4%, 95% CI: 4.6–8.1%) than in type 1 diabetics (5.5%, 95% CI: 3.2–7.7%) [6]. Diabetic foot syndrome is a serious late complication of diabetes that can put the patient at risk for infection, sepsis, and even amputation of varying degrees [7]. Treatment should aim to achieve full healing and keep the patient mobile by focussing on promoting wound healing and treating or compensating for the underlying disease that is the causative factor [8,9]. It was proven that patients with DFUs are mostly older people with a lower body mass index (BMI), longer diabetic duration, suffering from hypertension, diabetic retinopathy, and having a smoking history [6].

A common healing complication is long-term bacterial colonisation of the wound by various types of pathogens and early infection. Healing may also be compromised by tissue ischemia, interstitial oedema, or excessive mechanical stress on the wound [10]. DFUs have a high recurrent rate that varies widely in different regions, e.g., in Europe, the recurrence rate is 24.9% per person-year (95% CI, 20.0–29.7%) [11]. Thus, DFU is also considered a severe public health issue due to devastating complications and the high risk of amputations [12]. Management of wound infection should reflect the clinical status of the non-healing wound. Treatment of infection should not be influenced by microbial culture results alone. A generalised indication for oral or parenteral antibiotic therapy based solely on the presence of bacteria at the base of the wound is not an appropriate approach. Such practice is potentially dangerous and may lead to the emergence of polyresistant microbial strains and ultimately increase the cost of treatment [13,14]. The number of bacteria in the wound bed varies from contamination to invasive infection, and often, a biofilm is present [15]. One option to reduce the bioburden is autolytic debridement using medical grade honey (MGH) [16]. The therapeutic value of honey has been documented in the oldest medical literature, and it has been known since antiquity that honey has antimicrobial effects and promotes wound healing [17]. Various cultures have used honey as a traditional medicine for centuries to treat different disorders [18]. In contrast to regular honey from a beekeeper or a grocery store, MGH follows strict criteria to ensure the quality of the honey and its safety and efficacy for medical applications [19]. MGH is carefully selected, organic honey, proven to be free of pollutants, such as herbicides, pesticides, heavy metals, and antibiotics, gamma-irradiated to ensure it is free from harmful endospores, meets strict production and storage standards, complies with legal and safety regulations, as well as physicochemical criteria [19]. MGH exerts several biological processes, including antimicrobial, antioxidant, anti-inflammatory, and immunomodulatory activities, without harming the newly formed granulation tissue [19,20]. MGH consists of roughly 200 different compounds, mainly carbohydrates (80–85%) and water (15–17%), but also small amounts of amino acids, proteins, enzymes, essential minerals, vitamins, and various phytochemicals. Of the latter, flavonoids and phenolic acids are likely the most biologically active [21]. The exact molecular mechanisms of MGH and its constituents in these biological processes are extensively described in two recent review publications [22,23]. It is known that MGH is a good option for DFU treatment. Still, there is not enough evidence and articles about the effect of MGH on infected chronic DFUs, but some evidence already exists that supports its use [16,24,25,26].

Our case series study aimed to assess the effect of MGH on wound healing in patients with infected DFU. The tested MGH formulation has been known for 20 years as effective in wound treatment [27,28,29].

## 2. Results

### 2.1. Case 1

**Figure 1:**
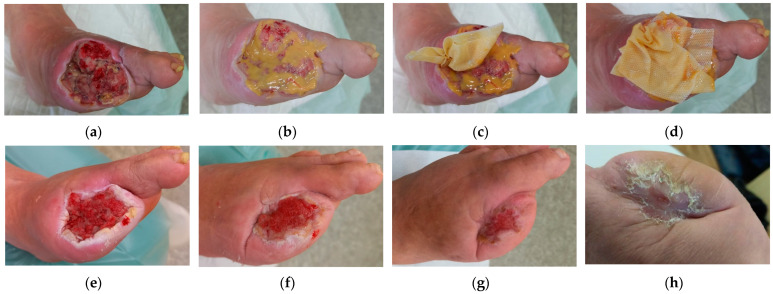
Case 1: Wound progression of a diabetic foot ulcer on the left foot. (**a**) Local finding at the initial examination, Day 0. (**b**) Example of L-Mesitran^®^ Ointment application on the wound bed. (**c**) Example of L-Mesitran^®^ Tulle application in the cavity. (**d**) Example of L-Mesitran^®^ Tulle application on the wound bed. (**e**) Follow-up examination on Day 30. (**f**) Follow-up examination on Day 66. (**g**) Follow-up examination on Day 102. (**h**) Complete wound healing on follow-up examination at Day 140.

A 63-year-old male patient presented with a DFU on the left foot following the amputation of digit I (Figure 1a). Upon presentation, the wound dimensions were 6 cm in length, 6 cm in width, and ranging from 5 cm to 1.5 cm in depth. The wound consisted mainly of roughly 60% of granulation tissue and 40% of slough. Medium levels of exudate (thin/watery) were produced. The wound edge was undermined at a 10 o’clock position and a depth of 5 cm. Periwound skin was without pathological signs. Local signs of infection included local warmth, increased exudate, delayed healing, malodour, and pocketing. Initially, the daytime pain level was of the value of one, and during treatment, it was one (diabetic neuropathy) A swab was performed upon presentation (Day 0) in which the following microorganisms were detected: (1). *Streptococcus dysgalactiae* massively with sensitivity to Penicillin, Erythromycin, Clindamycin, Tetracycline, Trimethoprim+sulfonamid, Bacitracin, Ciprofloxacin and Chloramphenicol, (2). *Pseudomonas aeruginosa,* with sensitivity to Gentamicin and Ciprofloxacin, (3). *Burkholderia multivorans* with sensitivity to Trimethoprim+sulfonamid and Neomycin, (4). *Staphylococcus aureus* with sensitivity to Cefoxitin, Trimethoprim+sulfonamid, Norfloxacin, Mupirocin, Neomycin, Bacitracin, Gentamicin, Chloramphenicol and Fusidic acid, and (5). *Escherichia coli* with sensitivity to Ampicillin, Aminopenicillin, Cefuroxime, Trimethoprim+sulfonamid, Cefpodoxime, Neomycin, Gentamicin, Ciprofloxacin, and Chloramphenicol.

It was decided to locally treat the DFU with L-Mesitran^®^ Ointment (MGH) (Figure 1b) on the inside of the lesion and L-Mesitran^®^ Tulle (MGH) at the pocketing (Figure 1c) and L-Mesitran^®^ Tulle (Figure 1d) to ensure the contact to the wound bed. Resposorb^®^ (superabsorbent dressing) was applied as a secondary dressing. The patient was instructed to perform the wound dressings at home at 48 h intervals for the first month. Local warmth, exudate, and malodour resolved and disappeared after 14 days of treatment. Subsequent examination on day 30 showed the wound dimensions decreased to 4 cm in length, 5 cm in width, and 0.8 cm in depth, and the undermining disappeared (Figure 1e). The wound consisted of 80% of granulation tissue and 20% of slough. Pain levels gradually decreased to zero, both during daytime and during treatment (diabetic neuropathy). Periwound skin was presented without pathological signs. A swab confirmed the elimination of *Burkholderia multivorans*, *Staphylococcus aureus*, and *Escherichia coli*, the continued presence of *Streptococcus dysgalactiae*, and *Pseudomonas aeruginosa*, and new contamination with *Enterobacter cloacae,* with sensitivity to Trimethoprim+sulfonamid, Cefpodoxime, Neomycin, Gentamicin, Ciprofloxacin and Chloramphenicol. Due to the positive evolution of the healing processes, the dressing change interval was extended to every three days. Topical treatment with excessive amounts of L-Mesitran^®^ Ointment increased the occurrence of maceration. The patient was instructed to use a smaller amount of ointment. Subsequent examination on Day 66 showed that the wound dimensions upon presentation were 3 cm in length, 4 cm in width, and 0.5 cm in depth (Figure 1f). The wound consisted of 95% of granulation tissue and 5% of slough. Pain levels remained at zero during daytime and during treatment. There were no signs of maceration or symptoms of infection at the wound edges. A new swab confirmed the elimination of *Streptococcus dysgalactiae*, the continued presence of *Enterobacter cloacae*, and *Pseudomonas aeruginosa*, and new contamination with *Enterococcus faecalis* with sensitivity to Ampicillin, Nitrofurantoin, Bacitracin and Chloramphenicol. The wound showed signs of healing but still produced positive swab examination on the presence of microorganisms; therefore, the use of L-Mesitran^®^ Ointment (due to microorganisms) with L-Mesitran^®^ Tulle was continued to ensure direct contact with the wound bed and Resposorb^®^ as a secondary dressing. Subsequent examination was performed on Day 102, and the wound dimensions upon presentation were 1.5 cm in length, 2 cm in width, and 0.1 cm in depth (Figure 1g). The wound consisted of 95% of epithelisation tissue and 5% of granulation. A swab confirmed further resolution of microorganisms with only *Enterococcus faecalis* remaining. Due to the positive evolution of the healing and limited colonisation, L-Mesitran^®^ Soft (MGH) was applied to the wound, and L-Mesitran^®^ Foam was used as a secondary dressing. The dressing change interval was extended to every four days. The subsequent examination on Day 140 showed that the wound was completely healed with MGH monotherapy without complications (Figure 1h). Due to the patient’s COVID-19 positivity, the photo was taken at home. We assume that the COVID-19 infection did not influence the healing process.

### 2.2. Case 2

**Figure 2:**
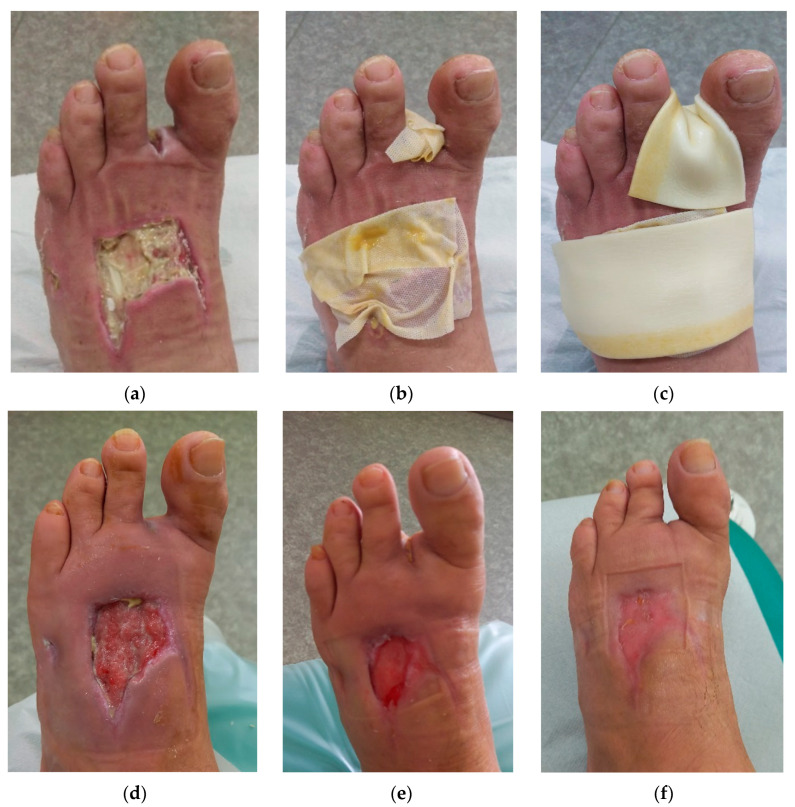
Case 2: Wound progression of two diabetic foot ulcers on the left foot (on the instep and after amputation of digit II). (**a**) Local finding at the initial examination, Day 0. (**b**) Example of L-Mesitran^®^ Tulle application on the wound bed. (**c**) Example of L-Mesitran^®^ Foam as a secondary dressing. (**d**) Follow-up examination on Day 42. (**e**) Follow-up examination on Day 79. (**f**) Complete wound healing on follow-up examination on Day 118.

A 49-year-old male patient presented with two DFUs on the left foot (Figure 2a). The first ulcer was on the instep and the second ulcer followed the amputation of digit II. Upon presentation (Day 0), the initial dimensions of the first ulcer were 7 cm in length, 6 cm in width, and 1.5 cm in depth, consisting mainly of 95% of sloughy and 5% of granulation tissue with medium levels of exudate (cloudy). The dimensions of the second ulcer were 2 cm in length, 2 cm in width, and 1 cm in depth, consisting of 20% of sloughy and 80% of granulation tissue with low levels of exudate (thin/watery). Local signs of infection included local warmth, increased exudate, delayed healing, and malodour. There were no pathological signs at the wound edge and periwound skin. Initially, the daytime pain level was of the value of one, and during treatment, it was one (diabetic neuropathy). A swab confirmed the presence of only *Enterococcus faecalis* with sensitivity to Ampicillin, Nitrofurantoin, Norfloxacin, Bacitracin, Gentamicin, Ciprofloxacin, and Chloramphenicol.

Based on the above findings, L-Mesitran^®^ Ointment and L-Mesitran^®^ Tulle (Figure 2b) were applied and covered with L-Mesitran^®^ Foam (Figure 2c) as a secondary dressing. The patient was instructed to perform wound dressings at home at 48 h intervals for the first month. Exudate, malodour, and pain resolved and disappeared after 14 days of treatment. During the next examination, on Day 42, the wound dimensions were 6 cm in length, 4.5 cm in width, and 1 cm in depth (Figure 2d). The wound consisted of 90% of granulation tissue and 10% of slough. The second ulcer dimensions upon presentation were 0.5 cm in length, 0.5 cm in width, and 0.5 cm in depth. The wound consisted of 95% of granulation tissue and 5% of slough. Low levels of exudate (thin/watery) were produced. A swab was performed in which the same pathogen was detected. Due to the positive evolution of the healing, the dressing change interval was extended to every three days. Local treatment was applied with L-Mesitran^®^ Tulle as a primary dressing and L-Mesitran^®^ Foam as a secondary dressing. During the next examination, on Day 79, the wound dimensions upon presentation were 4 cm in length, 2 cm in width, and 0.5 cm in depth, and consisting of 100% of granulation tissue (Figure 2e). The second ulcer dimensions were healed. There were no signs of inflammation. A swab confirmed that *Enterococcus faecalis* was still present. Due to the positive healing process, L-Mesitran^®^ Soft was applied to the wound bed, and L-Mesitran^®^ foam was applied as a secondary dressing. The dressing change interval was extended to every four days. After 118 days, the wound was completely healed with MGH monotherapy without complications (Figure 2f).

### 2.3. Case 3

**Figure 3:**
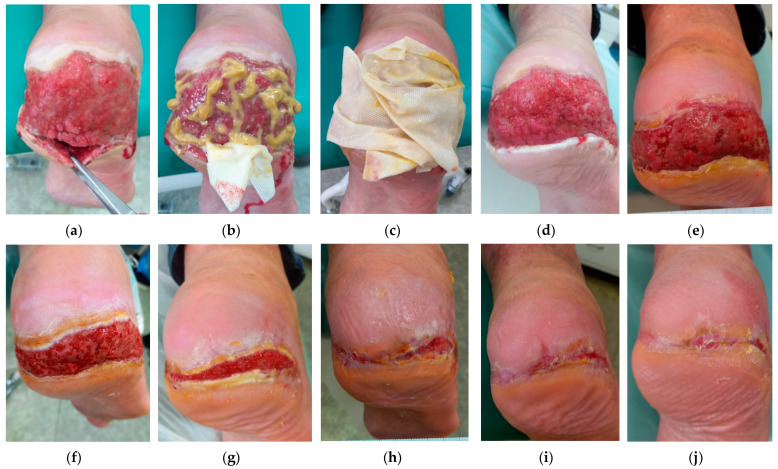
Case 3: Wound progression of a diabetic foot ulcer on the right heel. (**a**) Local finding at the initial examination, Day 0. (**b**) Example of L-Mesitran^®^ Ointment and L-Mesitran^®^ Tulle application at the pocketing. (**c**) Example L-Mesitran^®^ Tulle application on the wound bed. (**d**) Follow-up examination on Day 27. (**e**) Follow-up examination on Day 69. (**f**) Follow-up examination on Day 96. (**g**) Follow-up examination on Day 146, 14 July 2021. (**h**) Follow-up examination on Day 174. (**i**) Follow-up examination on Day 200. (**j**) A follow-up picture on Day 334 showed complete healing.

A 68-year-old male patient presented with a DFU on the right heel (Figure 3a). Upon presentation (Day 0), the wound dimensions were 8 cm in length, 18 cm in width, and ranging from 6 cm to 1 cm in depth (deeper 6 than cm at 6 o’clock). The wound consisted of roughly 70% of granulation tissue and 30% of slough and produced medium levels of exudate (thin, watery). Local signs of infection were delayed healing, pain, exudate, malodour, and pocketing. The daytime pain level was five and procedural pain was eight. There was hyperkeratosis and maceration on the periwound skin and wound edges. A swab confirmed the presence of (1) *Streptococcus agalactiae* with sensitivity to Penicillin, Trimethoprim+sulfonamid, Bacitracin, Ciprofloxacin, and Chloramphenicol, (2) *Alcaligenes faecalis,* with sensitivity to Trimethoprim+sulfonamid, Neomycin, Gentamicin, Ciprofloxacin and Chloramphenicol, and (3) *Morganella morganii* with sensitivity to Trimethoprim+sulfonamid, Cefpodoxime, Neomycin, Gentamicin, Ciprofloxacin, and Chloramphenicol.

Based on these findings, L-Mesitran^®^ Ointment (Figure 3b) was applied on the wound bed, and L-Mesitran^®^ Tulle was applied in the pockets (Figure 3b) and on the wound bed (Figure 3c) to ensure contact to the wound bed. Resposorb^®^ was applied as a secondary dressing. The patient was instructed to perform wound dressing changes at home at 48 h intervals. Pain, exudate, and malodour resolved after 14 days of local treatment. Pain levels gradually decreased, and after 14 days of treatment, the pain was tolerated (in the daytime, the pain level was zero, and during treatment, it was zero). The depth of pocketing also decreased. On Day 27, the wound dimensions were 6 cm in length, 16 cm in width, and 0.8 cm in depth (Figure 3d) with no signs of pocketing. The wound consisted of 98% of granulation tissue and 2% of slough, and the area of maceration and hyperkeratosis decreased. A swab was performed in which the same microorganisms were detected as in the previous swab. The secondary dressing was changed to L-Mesitran^®^ foam due to decreased levels of exudate and dressing changes were extended to every three days. During the next examination on Day 69, the wound dimensions upon presentation were 5 cm in length, 14 cm in width, and 0.5 cm in depth (Figure 3e). A swab confirmed that *Streptococcus agalactiae* was eliminated and the other two strains remained present. The change interval was extended to every four days. The next examination, on Day 96, showed that the wound dimensions upon presentation were 4 cm in length, 11 cm in width, and 0.5 cm in depth (Figure 3f). The wound consisted of 95% of granulation tissue and 5% of slough with low levels of exudate (thin/watery). Wound edges were hyperkeratotic. A swab detected four microorganisms: (1) *Enterococcus faecalis* with sensitivity to Ampicillin, Nitrofurantoin, Norfloxacin, Bacitracin, Ciprofloxacin, and Chloramphenicol, (2) *Staphylococcus aureus* with sensitivity to Oxacillin, Trimethoprim+sulfonamid, Ciprofloxacin, Mupirocin, Neomycin, Bacitracin, Gentamicin, Chloramphenicol, and Fusid acid, (3) *Acinetobacter baumannii,* with sensitivity to Trimethoprim+sulfonamid, Neomycin, Gentamicin, and Ciprofloxacin, and (4) *Morganella morganii*, with sensitivity to Trimethoprim+sulfonamid, Neomycin, Gentamicin, and Ciprofloxacin. During the next examination, on Day 146, the wound dimensions upon presentation were 1.5 cm in length, 8 cm in width, and 0.3 cm in depth (Figure 3g). The wound bed, wound edge, and periwound skin showed no signs of inflammation, and there were low levels of exudate. The patient did not report any pain. A swab confirmed the elimination of *Morganella morganii* and the remaining of the three other pathogens (*Enterococcus faecalis*, *Staphylococcus aureus*, and *Acinetobacter baumanii*). During the next examination, on Day 174, the wound dimensions upon presentation were 1 cm in length, 5 cm in width, and 0.3 cm in depth (Figure 3h). On Day 200, the wound dimensions were 0.5 cm in length, 0.5 cm in width, and 0.1 cm in depth (Figure 3i). The patient did not come for the next examinations because he was ill for the next three months. According to a teleconference (Skype), the wound healed, and there was no sign of inflammation. A follow-up picture after 334 days showed complete healing without complications (Figure 3j). The patient did not properly follow the recommendations for limb relief (armpits and walker boots) and the use of gloves when changing the local dressing.

### 2.4. Case 4

**Figure 4:**
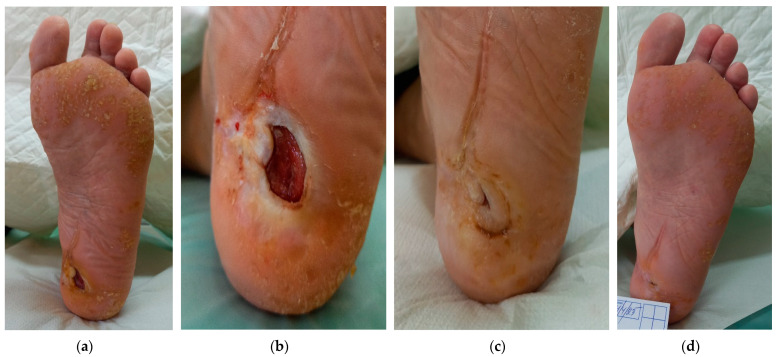
Case 4: Wound progression of a diabetic foot ulcer on the left heel. (**a**,**b**) Local finding at the initial examination, Day 0. (**c**) Follow-up examination on Day 30. (**d**) Complete wound healing on follow-up examination on Day 56.

A 63-year-old male patient presented (Day 0) with a DFU on the left heel (Figure 4a,b). The wound dimensions were 3 cm in length, 3 cm in width, and a depth up to 2 cm. The wound consisted of roughly 60% of granulation tissue and 40% of slough with medium levels of exudate (thin/watery). The wound edge was undermining at the 11 o’clock position and a depth of 1 cm and there was hyperkeratosis at the periwound skin. Local signs of infection were delayed healing, malodour, exudate, and pocketing. There was a tinea pedis on the plantar surface of the foot. Initially, the daytime pain level was of the value of one, and during treatment, it was one (diabetic neuropathy). A swab detected (1) *Staphylococcus aureus* with sensitivity to Oxacillin, Clindamycin, Trimethoprim+sulfonamid, Mupirocin, Neomycin, Bacitracin, Chloramphenicol, and Fusidic acid, (2) *Enterococcus faecalis,* with sensitivity to Ampicillin, Nitrofurantoin, Norfloxacin, Bacitracin, Ciprofloxacin, and Chloramphenicol, and (3) *Escherichia coli* with sensitivity to Ampicillin, Aminopenicillin, Cefuroxim, Trimethoprim+sulfonamid, Cefpodoxim Neomycin, Gentamicin, Ciprofloxacin, and Chloramphenicol.

Based on these findings, L-Mesitran^®^ Ointment was applied inside the lesion and L-Mesitran^®^ Tulle was applied at the pocketing to ensure contact with the wound bed. HydroTac Concave^®^ (foam with the gel) was applied as a secondary dressing. The patient performed wound dressing changes at home at 48 h intervals. Malodour and pocketing resolved and disappeared after 14 days of treatment. Subsequent examination realised on day 30 showed that the wound dimensions upon presentation were 0.5 cm in length, 0.5 cm in width, and 0.8 cm in depth (Figure 4c). The wound consisted of 90% of granulation tissue and 10% of slough, and the undermining disappeared. Pain levels gradually decreased, and after 30 days of treatment, the pain was tolerated and scored zero during both daytime and treatment (diabetic neuropathy). There were no pathological signs on the periwound skin. A swab confirmed the elimination of Staphylococcus aureus and the remaining of the other two pathogens. Due to the positive evolution of the healing processes, the dressing change interval was extended to every three days. On Day 56, the wound was completely healed without complications (Figure 4d). Removal of the hyperkeratosis caused small wounds in and around the scar.

### 2.5. Case 5

**Figure 5:**
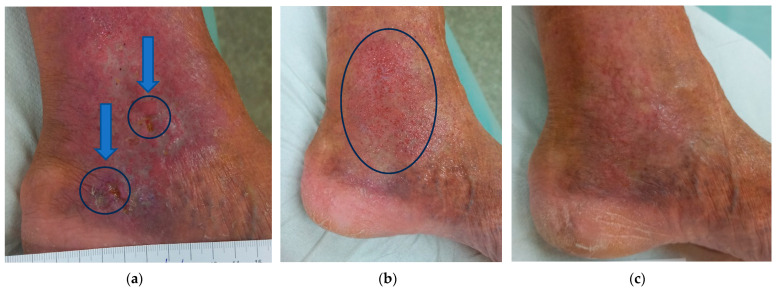
Case 5: Wound progression of two DFUs on the left foot below the medial ankle. (**a**) Local finding at the initial examination, Day 0. Blue circles indicate the wounds. (**b**) Follow-up examination on Day 54—complete original wound healing, but the appearance of the microbial colonisation (indicated by a blue oval) remained despite a negative swab as well as skin irritation. (**c**) Follow-up examination on Day 103 with a normal appearance of the skin.

A 65-year-old male patient presented (Day 0) with two DFUs on the left foot below the medial ankle (Figure 5a). The wound dimensions were 0.8 cm in length, 0.5 cm in width, and 0.8 cm in depth for Wound 1 (top) and 1.8 cm in length, 0.5 cm in width, and 0.8 cm in depth for Wound 2 (bottom). The wounds consisted of roughly 80% of granulation tissue and 20% of slough and produced medium levels of exudate (thin/watery). There were no pathological signs on the wound edge and the periwound skin. Local signs of infection included increased pain, delayed healing, exudate, and malodour. At the initial time point, the daytime pain level was of the value of five, and during treatment, the value was six. A swab detected *Staphylococcus aureus* (massively) with sensitivity to Oxacillin, Clindamycin, Trimethoprim+sulfonamid, Mupirocin, Neomycin, Bacitracin, Gentamicin, Chloramphenicol, and Fusidic acid.

Based on these findings, local treatment with L-Mesitran^®^ Ointment as a primary dressing and L-Mesitran^®^ Foam as a secondary dressing was initiated. The patient performed wound dressings at home at 48 h intervals. Malodour and pain (VAS 3) resolved and disappeared after 14 days of treatment. On Day 54, the original wounds healed, but cranially, above the original wound, microbial colonisation appeared, still present (Figure 5b), although the bacterial swab was negative. The chosen local treatment was L-Mesitran^®^ Foam. The dressing change interval was extended to every three days. The subsequent examination on Day 103 showed that the appearance of the skin was normal (Figure 5c). No complications were noticed during the treatment with MGH.

### 2.6. Overview of Wound Healing Time

**Figure 6:**
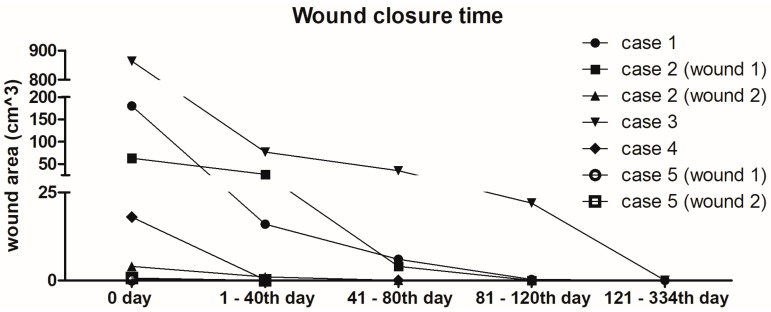
Graphical representation of wound closure time. The *y*-axis is split into three segments because of the large variety in wound areas.

Wound healing time is an important endpoint as it represents duration of time when patients suffer from their wounds. However, due to unique patient and wound characteristics, such as age, comorbidities, environmental factors, wound size, presence of infection, etc., the healing time is affected. A graphical representation of the wound healing times of the included patients clearly illustrates the large variability due to these personal factors (Figure 6). Since the patients were outpatients and the days of follow-up varied, we summarised the follow-up data for all patients into several periods until complete wound closure. When the wound was closed during the consultation, it is possible the wound was closed at an earlier time point. For example, for Case 5, the first follow-up consultation was after 56 days at which the wounds were healed; however, they might have already healed days or weeks earlier. The wound closure occurred in all treated patients. As can be deduced from Figure 6, the smaller the wound area in cm^3^, the shorter the wound closure time.

### 2.7. Influence of MGH on Glycaemia Levels

**Figure 7:**
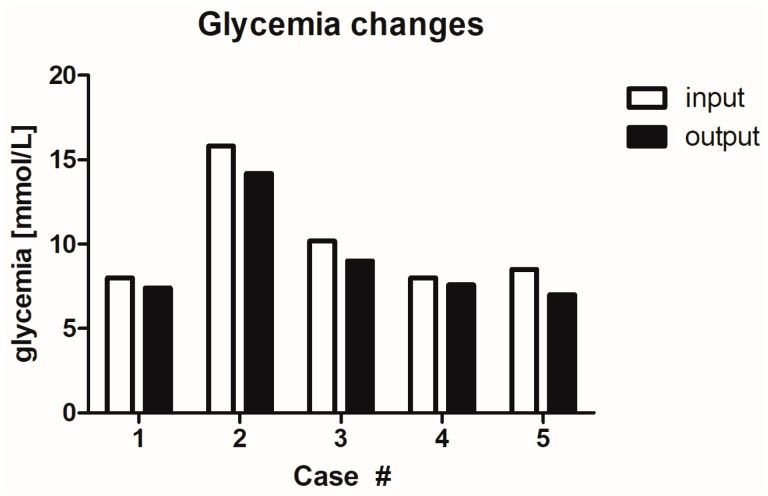
Glycaemia levels during wound care treatment. Start of MGH treatment (input) and after complete healing (output).

**Figure 8:**
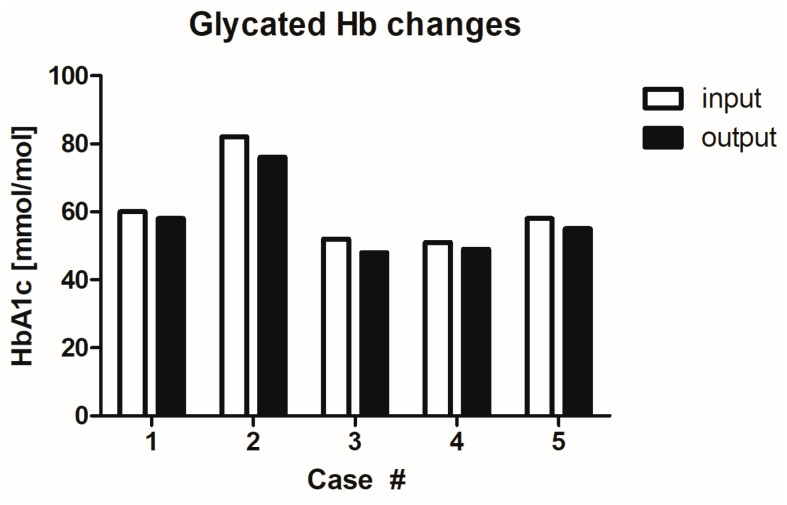
Glycated haemoglobin (Hb) levels during wound care treatment. Start of MGH treatment (input) and after complete healing (output).

An important question that may arise is whether the honey, or specific sugars in honey, can elevate the blood glucose levels of diabetics. Therefore, the blood glucose levels (glycaemia) and glycated haemoglobin levels in all patients were measured before the start of MGH treatment (input) and after the complete healing of the wound (output) (Figure 7 and Figure 8). All patients experienced a reduction in glycaemia levels from 10.1 ± 3.3 mmol/L (mean ± SD) to 9.0 ± 3.0 mmol/L (mean ± SD), indicating an average reduction of 10.4%, range 5.0–17.6%. The glycated haemoglobin levels also reduced in all patients, from 60.6 ± 12.6 (mean ± SD) to 57.2 ± 11.3 (mean ± SD), indicating an average reduction of 5.5 %, range 3.3–7.7%) during MGH treatment.

## 3. Discussion

In all five cases included in the study, the healing process was positively influenced by cleaning the wound bed and gradually eliminating the odour and pain until disappearance. Furthermore, there was a reduction in the exudation. Despite the demonstrated microbial load in the wounds, using swabs and cell culture examination, antibiotic treatment was unnecessary. MGH suppressed the signs of local infection and gradually disappeared with the increasing length of material application. In fact, despite the presence of a variety of virulent and opportunistic pathogens in the wound bed (*Streptococcus dysgalactiae*, *Pseudomonas aeruginosa*, *Burkholderia multivorans*, *Staphylococcus aureus*, *Escherichia coli, Enterobacter cloacae*, *Enterococcus faecalis*, *Streptococcus agalactiae*, *Alcaligenes faecalis*, *Morganella morganii*, and *Acinetobacter baumannii*), antibiotic treatment was not necessary to completely resolve the infections. The median wound healing time to complete healing was 150 days. However, it should be noted that in Case 3, the wound healed after 334 days, which we attribute to the patient’s noncompliance with the recommendations for leg offloading during wound healing. One patient (Case 1) had COVID-19 during the study, which may have also affected the wound healing duration. After proper instruction, patients were able to perform wound dressings on their own or with the help of family members at home, which was considered to be easy. At the start of the treatment, dressing changes were recommended every two days, and this was gradually extended to every three or four days with positive evolution of the wound. No adverse effects or allergic reactions related to the topical MGH treatment were observed. Since MGH consists of roughly 80% of sugars, it was investigated whether topical application affects the sugar levels in diabetic patients. Despite the outflow mechanism of action by MGH osmotic activity and the product not being absorbed by the body, it is important to exclude the effects of MGH on blood glucose levels in diabetics. A positive effect of topical treatment with MGH in patients with diabetic foot syndrome was shown. In all patients, glycated haemoglobin and glycaemia levels gradually decreased. This is likely the result of eliminating wound-related inflammatory mediators, which have a role in inducing insulin resistance and promoting hyperglycaemia [30,31]. In addition, the changes in blood glucose levels may also be attributed to the appropriate education about the need for lifestyle changes and a healthy diet as part of the treatment process. Thus, MGH does not negatively influence blood glucose levels. In line, a retrospective study among 36 diabetic patients showed no effect on blood glucose levels when their wounds were treated with MGH [32].

The financial cost of wound care in developed countries is around 1–4% of total healthcare expenditure [15,33]. It is expected that the number of patients suffering from non-healing wounds will continue to increase due to increasing age and the growing number of comorbidities such as obesity, diabetes mellitus, and cardiovascular diseases, thus increasing their economic impact [2,10,34,35]. Especially chronic wounds such as DFUs represent a significant clinical, social, and economic problem [36,37]. Three factors determine cost efficacy in wound care: (1) healing time, (2) frequency of dressing changes, and (3) complications [38,39]. According to the results of the study, local treatment with MGH materials was considered cost effective because patients were able to perform wound dressings by themselves at home, the products were affordable, and wounds healed in a relatively short time (related to patient comorbidities and the presence of pathogens at the wound bed) without complications, and antibiotic and analgesic treatment was not needed.

The different pro-healing activities of MGH resonated in the study. MGH favourably promoted autolytic debridement, led to wound bed clearance, and positively promoted the formation of granulation and epithelial tissue from the wound margins and edges. This is in line with previous studies, where the autolytic debridement and wound bed clearance were attributed to the formation of a moist wound bed, its acidification, osmotic activity, and the oxygenation of the wound bed environment [14,15,40]. The wound healing progression and time to complete healing is in line with other clinical studies investigating the efficacy of MGH on chronic infected wounds [16,41,42]. This has also been attributed to the range of pro-healing mechanisms of MGH, including its anti-inflammatory, antioxidant, antimicrobial, and anti-biofilm activities.

The prevalence of biofilms in non-healing wounds is estimated to be approximately 60% [43,44]. However, higher numbers of 65, 80, and up to 100% are also reported, making biofilms an essential factor to consider in wound healing [45,46]. Biofilms are microbial aggregates encased by their self-produced matrix which harnesses these pathogens from host immunological responses and antimicrobial and antiseptic treatments, and thus increases bacterial resistance [15,47,48]. Biofilm in wounds often causes their recurrence [46]. Interestingly, MGH can eradicate biofilms [49]. In our study, we verified the positive effect of topical MGH material on virulent and opportunistic pathogens. Positive effects on the elimination of pathogens *Staphylococcus aureus* (including MRSA), *Pseudomonas aeruginosa,* and *Streptococcus*, among others, have been confirmed by others [16,20,41,49,50]. Due to its antimicrobial mechanisms, including acidic pH, osmotic activity, and slow release of hydrogen peroxide during the catalysation of glucose by glucose oxidase, MGH is effective against a wide range of pathogens, including multi-resistant bacteria, fungi, and viruses [13,22,51]. Moreover, the use of MGH in wound management can reduce the use of antibiotics and topical antiseptics [40]. Importantly, there is no risk of developing resistance toward MGH [13,16,52]. In our study, all patients had local signs of infection, and microbial burden in the wound bed was demonstrated in all (n = 5) patients. No patient required antibiotic treatment. The authors reached the same conclusion in a previously published study [53], verifying the antimicrobial effects of MGH in their study conducted on nine patients. Interestingly, a previous study on surgical wounds among 766 patients demonstrated that MGH was as effective in preventing surgical site infections as antibiotics combined with povidone–iodine, while MGH also enhanced healing time [52]. MGH has antimicrobial, antioxidant, and anti-inflammatory properties and is therefore ideal for treating infected wounds [14,34,54].

Eliminating the negative symptoms accompanying non-healing wounds is important for improving the patient’s QoL [4]. Pain is not only a sensory but also an emotional component associated with anxiety, depression, aggression, feelings of threat, hopelessness, and loss of motivation [55,56]. Patients with wounds also suffer from procedural pain [57]. Stress, anxiety, and fear experienced during wound care increase cortisol levels, which have a negative effect on wound healing [58,59]. In an RCT with 40 patients, wounds were treated with honey (intervention group) or conventional dressings (controls). After three months, 16 (80%) individuals in the intervention group had their wounds completely healed, compared with only six (30%) patients in the control group. MGH resulted in faster healing, wound size reduction, and lower pain intensity [60]. In another study with 25 patients having chronic venous leg ulcers, the average wound area decreased significantly [18]. Eighteen patients (72%) experienced a reduction in reported pain levels, while five patients (20%) experienced the same pain level throughout the study. Overall satisfaction with the honey treatment was positive in 80% of patients. Only two patients demonstrated poor tolerability due to pain-related problems at the ulcer site [18]. A prospective observational study on 20 patients with chronic pressure ulcers with spinal cord injury showed that all swabs were free of bacterial growth one week after topical treatment with MGH [40]. A total of 18 patients (90%) showed complete wound healing after four weeks. No adverse effects of treatment with MGH were noted [40]. In a case series (n = 6) with non-healing wounds, MGH initiated healing, infection was controlled, and QoL was strongly improved (malodour, exudate levels, and pain swiftly decreased) [41]. All wounds healed relatively quickly, considering the severity of the wounds and the general patient health [41]. In all the cases in our study, the procedural pain was reduced or eliminated during the procedure. Patients did not require the use of analgesic treatment. This may be due to the appropriate choice of local material, which produced a sufficiently moist environment to prevent adherence to the wound bed and the development of procedural pain during dressing changes and between rebandaging the wounds.

Wound odour is another unpleasant symptom of infection in non-healing wounds that has a negative impact on patient psychological status and is associated with abundant exudate production, and both of these factors can lead to social isolation [16]. Malodorous wounds can be distressing for patients and their families, negatively impacting QoL outcomes. The odour can also be perceived by medical staff [61]. In our study, there was a reduction in odour and exudate immediately after the first dressing change. Wound odour is produced by bacteria that metabolise serum, tissue proteins, and dead cells, all of which produce amino acids and an unpleasant odour [62,63]. Glucose in topical materials with MGH offers an alternative odourless substrate for these bacteria, thus eliminating the odour [16,49,62,63]. Furthermore, the antimicrobial activity of honey reduces the number of bacteria in the wound, thereby reducing odour [27,62]. This ability is most apparent within 24 h of applying honey to the wound [26]. Also, in our study, patients reported a reduction in odour after the first wound dressing (the shortest dressing interval was two days).

Topical dressings with MGH were able to maintain optimal moisture in the wound. Only in patient Case 1 the maceration of the wound edges (0.3 cm from the wound edge) was observed. We learned that the patient applied a large amount of L-Mesitran^®^ Ointment to the wound at home. The maceration did not occur again after proper instruction to apply a smaller amount of L-Mesitran^®^ ointment. The high sugar content of local materials with MGH attracts lymphatic fluid and wound exudate from the tissue. This process, together with the anti-inflammatory activity of MGH, subsequently reduces the incidence of swelling and pain [16]. Due to favourable healing, extending the interval between dressings to three or four days was possible. Longer intervals between dressings were not recommended due to the possible occurrence of biofilm. Non-healing wounds represent an economic burden for all healthcare systems worldwide. Extending the time between wound dressings, reducing the healing time of wounds, not administering antibiotics, and allowing the performance of wound dressings at home (in a natural social setting or with the help of home care nurses) can significantly reduce costs [53]. In our study, MGH demonstrated a positive effect on the healing process and patient QoL. We believe that material with MGH should be used to treat non-healing wounds with signs of local infection. In the past, MGH materials were not recommended for patients with diabetes and manifestations of diabetic foot syndrome. In our case–control study, we verified that the use of MGH did not have a negative effect on glycated haemoglobin levels or glycaemia during healing. Our claim is consistent with other studies showing MGH local treatment in patients with diabetic foot syndrome does not increase blood glucose levels [16,32].

It is essential to emphasise that a consistent, comprehensive approach to the care of the overall patient condition contributed to the effect of local therapy. Despite the convincing efficacy of MGH for the treatment of infected DFUs in our case series and the findings being in line with other studies and our own clinical experiences, the number of cases presented is limited. Therefore, to further substantiate our findings, studies with a bigger sample size are encouraged, preferably in double-blinded randomized controlled trials comparing MGH with other therapies. However, the experimental setup of these studies needs to be carefully considered since wound and patient characteristics (cause, type, stage, dimensions, colonisation, presence of biofilms, previous treatments, age, health, nutrition, comorbidities, environmental factors, etcetera) are unique and hard to homogenously distribute.

## 4. Materials and Methods

### 4.1. Patient Selection

In a prospective observational study, case–control series, MGH was applied to a selected group of patients with non-healing wounds, particularly diabetic foot ulcers. The inclusion criteria were having a DFU with treatment lasting more than six weeks (internal control), having type 2 diabetes mellitus, glycated haemoglobin HbAc1 higher than 50 mmol/L, presence of local signs of inflammation, a positive swab culture indicating microorganisms in the wound bed, control examination within maximum 2 months (range 22–60 days), patient agreement—informed consent. The exclusion criteria were having an allergy to bee stings or MGH, the presence of systemic signs of inflammation, and patient disagreement to participate in the study.

A total of five male patients were included in our prospective case–control study (Table 1). The average age was 61.6 years (min. 49 and max. 68, with a median of 63 years). All patients were diagnosed with diabetic foot syndrome with the presence of non-healing wounds (DFU), with treatment lasting more than six weeks. Previous therapy consisted of iodine material, wound gel, ointment, solution, foam, and rapeseed. All patients showed local signs of wound infection, and signs of systemic infection were absent. The average assessment of pain intensity during the day reached 2.6 points, and procedural pain averaged 3.4 points according to VAS (0 to 10 points scale). Still, two patients had diabetic neuropathy. Therefore, the pain value was reduced.

### 4.2. Wound Care and Assessment Details

The basic treatment process was the same in all patients. Mechanical debridement of the wound bed, dressing with antiseptic solution (20 min), and application of protective spray on the area around the wound were performed. All the wounds were treated with MGH-based wound care products (full range of L-Mesitran products, manufactured by Theo Manufacturing, Maastricht, the Netherlands, www.mesitran.com (accessed on 7 July 2023). The honey used in their products can come from different geographical regions and floral origin but always follow the strict criteria to qualify as MGH [19]. Moreover, other supplements added to the formulation such as vitamins C and E synergistically enhance the beneficial activities of MGH [49]. Patients performed mechanical debridement by themselves at home, which helped in wound hygiene and the healing process. The photo at the beginning and final status are presented. The first follow-up examination was performed after 14 days of established topical treatment. Wounds were assessed according to the “*Triangle of Wound Assessment*” designed for holistic wound assessment considering wound bed, wound edge and periwound skin [1]. In these assessment areas, other parameters are also evaluated to better assess a non-healing wound and start adequate local therapy. The wound bed parameter also assesses tissue type, exudate and infection. The tissue type assesses the colour that is present at the wound bed (black colour—necrotic, yellow colour—sloughy, red colour—granulation, pink colour—epithelisation). This colour spectrum is presented as a percentage of the wound and is based on subjective assessment by the treating wound care specialist. The wound edge is assessed by evaluating maceration, dehydration, undermining, and rolling. The periwound skin is assessed by evaluating maceration, excoriation, dry skin, hyperkeratosis, callus, and eczema. Signs of infection included a positive swab (obtained using the Levine technique), delayed healing, local warmth, exudate, pocketing, odour, and pain. The pain was assessed using a visual analogue scale (VAS; 0–10 points), both during the day and during procedural pain (during intervention—dressing changes).

The patients were informed about the study, and they all provided written consent to use their photos and data for publication, providing their anonymity was guaranteed. The World Medical Association’s Declaration of Helsinki principles were followed.

## 5. Conclusions

In our prospective observational study, case-control series, we confirmed in a group of five patients with diabetic foot syndrome that treatment with topical MGH positively affects the healing process in non-healing wounds. The application of MGH-containing dressing led to the activation of the healing process, debridement stimulation, and faster wound bed cleaning phase. MGH reduced odour and exudate secretion and maintained an optimal moist environment in the wound bed, thus aiding the cleansing phase of the healing process. Despite the presence of local signs of infection and the presence of virulent and opportunistic microorganisms, there was no need for antibiotic treatment and MGH eliminated all pathogens. Wound-related pain and procedural pain were significantly reduced, and there was no need to administer analgesic drugs. Local treatment of non-healing wounds with MGH dressings was easy for patients and their relatives. MGH treatment limited the financial costs of care, reduced the risk of complications, and fastened the healing process, together supporting its cost-effectivity. MGH can safely be used in diabetics; glycated haemoglobin decreased gradually due to the disappearance of local inflammation and a positive healing process. The reduction in symptoms significantly improved patient QoL with non-healing wounds. All wounds were healed. The topical application of MGH constitutes a qualitative alternative to antibiotic treatment when local signs of infection occur while improving the wound healing trajectory.

## Figures and Tables

**Table 1:** Overview of the presented cases.

Case#	Gender/Age (years)	Patient Characteristics	Wound Type and Location	Wound Age	Previous Treatments	Signs of Infection	Pain VAS (Score 1–10) Daytime/Procedural	Analgesic/Antibiotic Treatment	Baseline/Final glycated Haemoglobin	Baseline/Final Glycaemia
1	Male, 63	DM (oral antidiabetic drugs), diabetic gangrene, amputation of the big toe on the left foot, diabetic neuropathy, HT, obesity class III (BMI = 45), Charcot’s osteoarthropathy, phlegmon pedis, COVID-19	DFU at the left foot	2 months	Wound gel	Local warmth, exudate, delayed healing, malodour, pocketing	1/1 (diabetic neuropathy)	No/No	60/58 mmol/mol	8/7.4 mmol/L
2	Male, 49	DM (insulin therapy), diabetic gangrene, amputation of digit II on the left foot, diabetic neuropathy, HT, normal weight (BMI = 23.1), ischemic disease of the lower limbs second degree, chronic pancreatitis	DFUs at the left foot	6 weeks	Iodinated povidone solution	Exudate, delayed healing, malodour	1/1 (diabetic neuropathy)	No/No	82/76 mmol/mol	15.8/14.2 mmol/L
3	Male, 68	DM (oral antidiabetic drugs), HT, hemochromatosis, CVI, dyslipidaemia, obesity class II (BMI = 35.6), Parkinson’s disease, polyneuropathy lower limbs	DFUs at the right foot on the heel	6 weeks	Iodinated povidone solution	Increased pain, increased exudate, delayed healing, malodour, pocketing	5/8	No/No	52/48 mmol/mol	10.2/9
4	Male, 63	DM (insulin therapy), diabetic gangrene, diabetic neuropathy, overweight (BMI = 29.4)	DFUs at the left foot on the heel	6 months	Rapeseed, wound gel, foam	Delayed healing, malodour, pocketing	1/1 (diabetic neuropathy)	No/No	51/49 mmol/mol	8/7.6 mmol/L
5	Male, 65	DM (oral antidiabetic drugs), HT, CVI, gouty disease, obesity class I (BMI = 30.9)	DFUs at the left foot under the media ankle	6 weeks	Ointment, solution	Increased pain, delayed healing, malodour	5/6	No/No	58/55 mmol/mol	8.5/7.0 mmol/L

CVI—Chronic venous insufficiency; DM—diabetes mellitus type 2; HT—hypertension; VAS—visual analogue scale; baseline glycated haemoglobin—the level of HbAc1 at the beginning of the care/treatment with MGH; final glycated haemoglobin—the level of HbAc1 at the end of the care/treatment with MGH.

## Data Availability

The data supporting this study’s findings are available from the corresponding author upon reasonable request. All data relevant to the study are included in the article. The data are safely stored as requested by Czech legislation in a healthcare provider secured electronic system.
